# Organosolv Treatment of Red Grape Pomace for Effective Recovery of Antioxidant Polyphenols and Pigments Using a Ternary Glycerol/Ethanol/Water System under Mild Acidic Conditions

**DOI:** 10.3390/molecules29030563

**Published:** 2024-01-23

**Authors:** Maria Geropoulou, Elissavet Yiagtzi, Theodoros Chatzimitakos, Dimitrios Palaiogiannis, Dimitris P. Makris

**Affiliations:** Green Processes & Biorefinery Group, Department of Food Science & Nutrition, School of Agricultural Sciences, University of Thessaly, N. Temponera Street, 43100 Karditsa, Greecetchatzimitakos@uth.gr (T.C.);

**Keywords:** anthocyanin pigments, antioxidants, red grape pomace, organosolv treatment, polyphenols

## Abstract

The purpose of this investigation was (i) the development of a novel, green tertiary solvent system, composed of water, ethanol and glycerol, and (ii) the implementation of an organosolv treatment of red grape pomace (RGP) for the efficient production of polyphenol-containing extracts with enhanced antioxidant properties. The treatment developed was performed under mild acidic conditions, imparted by the addition of citric acid, and it was first evaluated on the basis of severity, establishing linear models that described the correlation between treatment performance and combined severity factors. To solicit treatment optimization, response surface methodology was implemented, considering solvent acidity and residence time as the treatment variables. The optimized treatment afforded maximum total polyphenol (166 ± 6 mg GAE g^−1^ DM), total pigment (4.4 ± 0.2 mg MvE g^−1^ DM) and total flavanol (31.5 mg CtE g^−1^ DM) yields and extracts with particularly enhanced antioxidant activity. This might be attributed to specific constituents with high antioxidant potency, such as catechin, determined in the extract using high-performance liquid chromatography. Thus, the treatment developed is proposed as a highly efficient process to generate RGP extracts enriched in polyphenolic compounds, with enhanced antioxidant activity. Such extracts might then be valorized as food additives, to provide antioxidant protection and/or pigmentation.

## 1. Introduction

The challenges that the world’s population is currently facing with respect to environmental pollution and bioresource depletion have reached an unprecedented level. The uncontrolled overexploitation of land and water resources, along with the ever-increasing waste biomass generation, have raised overwhelming concerns about linear economy models. On the contrary, bioeconomy and circular economy strategies are gaining wide acceptance as models with high prospects in sustainable development, ecosystem preservation and environmental protection. In this frame, waste biomass valorization to attain zero-waste agricultural and agriculture-based industrial production has become an imminent priority [1,2].

Grapes (*Vitis vinifera* spp.) are one of the largest fruit crops around the globe, and there exist approximately 2000 different grape varieties. According to the International Organization of Vine and Wine (OIV), in 2018, the world’s grape production mounted up to about 77.8 million tons, 57% of which was destined for winemaking [3]. Wine production is an industrial activity responsible for generating high amounts of waste, considering that, on average, almost 20–30% of the grapes used for wine production end up as by-products [4,5]. This waste material is mainly composed of pomace (skins and seeds), but also grape stems, and it is particularly rich in a spectrum of polyphenolic compounds, existing in a wide diversity of forms [6,7].

Over the last decades, there has been an enormous number of studies reporting on the polyphenolic composition of red grape pomace (RGP), owing to their unique and multiple beneficial biological properties. These properties may include, but are not limited to, activity against the onset of cardiovascular disorders, as well as chemopreventive, anti-inflammatory, antimicrobial and antioxidant activities [8]. Thus, by virtue of such an assortment of effects, RGP is regarded as one of the richest and most biopotent sources of polyphenolic substances. It is not surprising, therefore, that the development of technologies for recovering RGP polyphenols and pigments has been the subject of numerous investigations [9,10].

Biorefinery technologies engulf innovative tools and methodologies to convert the organic residues of agricultural practices and food processing into value-added and high-value-added products, such as energy, building block chemicals, food/cosmetic ingredients and/or pharmaceutical agents. The way forward in this direction may be paved by developing cutting-edge technologies, which would aim at generating compounds of high purity and stability, with minimal energy requirements and environmental risks. In this regard, the search for alternative solvents and the implementation of eco-friendly approaches for the effective recovery of bioactive phytochemicals is of particular importance [11,12].

To date, the development of organosolv processes, that is, the combination of organic solvents or water/solvent mixtures at relatively high temperatures, is dictated by the use of environmentally benign solvents to replace petroleum-based volatile organic ones. Ideally, a green solvent should be non-toxic, recyclable, inert, and available at low cost and with low vapor pressure. Thus, one of the most promising, green and food-grade liquids is glycerol, which was explicitly proposed as a solvent for natural compounds (polyphenols) extraction almost a decade ago; ever since, numerous methodologies pertaining to the extraction of bioactive substances have been developed, illustrating the usefulness and efficiency of glycerol in the recovery of polyphenolic substances [13]. Although a number of studies pertaining to the use of glycerol demonstrated its suitability for pertinent processes, an intrinsic drawback related to glycerol is its high viscosity, which may hinder effective solute (polyphenol) diffusivity and complicate further extract processing (e.g., filtration). Such an obstacle could be overcome by using glycerol as a mixture with water. In this case, problems in polyphenol extractability may arise from the high water polarity.

This being the case, the current project was undertaken to examine ternary mixtures of water/glycerol/ethanol as high-performance solvents for RGP organosolv treatment, with the aim of achieving increased polyphenol recovery. Treatments were assisted by mild acid catalysis, imparted by citric acid, which has been implicated in hydrolytic reactions [14,15], and could facilitate polyphenol liberation through cell wall disruption [16]. Treatment performance was evaluated by considering severity, and it was optimized by deploying response surface methodology. Finally, extract assessment was accomplished by determining the analytical polyphenolic profile and the antioxidant activity. As far as the authors are aware, such an RGP treatment for effective polyphenol recovery has not been reported heretofore.

## 2. Results and Discussion

### 2.1. Single-Factor Experimentation

**Figure 1:**
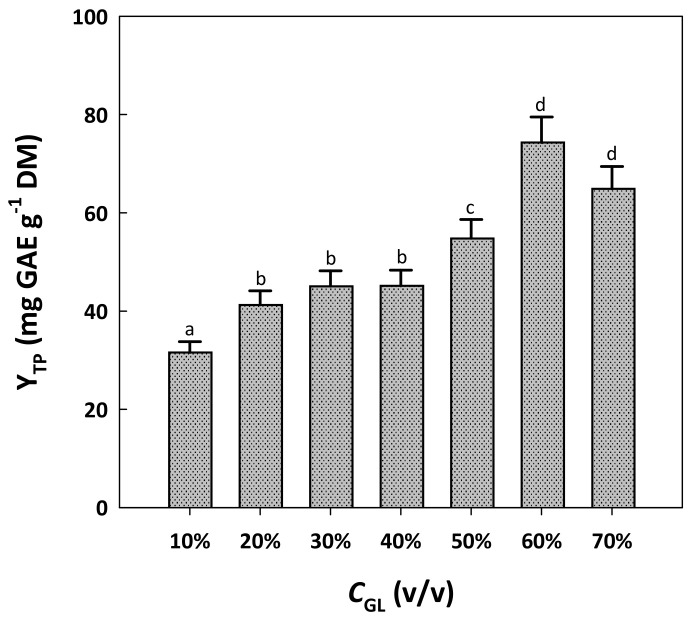
The effect of glycerol proportion in the solvent system tested on the yield in total polyphenols. In all cases, the solvent system contained a standard water proportion of 10% and varying proportions of ethanol. Treatments were accomplished at a constant temperature of 90 °C, for 180 min. Columns assigned with different letters (a, b, c, d) represent statistically different values (*p* < 0.05).

An earlier examination of RGP polyphenol extraction using water/glycerol mixtures showed that a solvent composed of 90% (*w*/*v*) glycerol could be very effective, yet this solvent system was particularly viscous and its handling difficult and problematic [17]. Based on this observation, the water content of the solvent systems tested was maintained constant at 10%, and the extraction efficacy was assayed by switching the proportion of ethanol/glycerol. Glycerol was not tested beyond 70%, to obtain extracts that could be readily centrifuged. Figure 1 depicts the yield in total polyphenols recorded as a function of glycerol concentration (*C*_GL_) in the mixtures tested.

The incorporation of glycerol at a level of 10% was inefficient, as this solvent system provided significantly low Y_TP_ (*p* < 0.05). Switching C_GL_ from 20 to 60% gave practically similar Y_TP_, but setting C_GL_ at 60% provided significantly higher Y_TP_ (*p* < 0.05). A further increase in C_GL_ to 70% did not favor higher Y_TP_. Therefore, the mixture composed of 10% water, 60% glycerol and 30% ethanol was chosen as the most efficacious.

**Figure 2:**
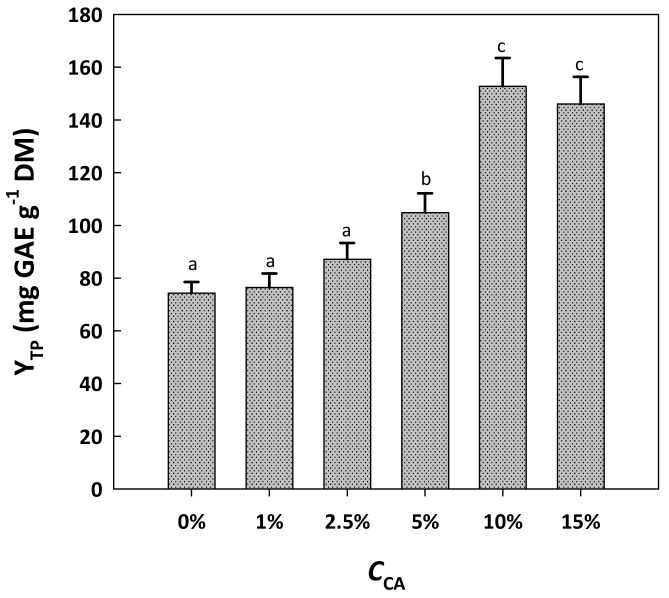
The effect of citric acid concentration (*C*_CA_) in the solvent system tested on the yield in total polyphenols. In all cases, the solvent system was composed of 10% water, 60% glycerol and 30% ethanol. Treatments were accomplished at a constant temperature of 90 °C for 180 min. Columns assigned with different letters (a, b, c) represent statistically different values (*p* < 0.05).

The next step in boosting the efficiency of the solvent system was the examination of the effect of citric acid addition. Thus, citric acid was added to the solvent at concentrations (*C*_CA_) varying from 1 to 15% (Figure 2). Up to *C*_CA_ of 2.5%, citric acid exerted a negligible effect on Y_TP_. However, at *C*_CA_ of 10%, the extraction yield was significantly enhanced (*p* < 0.05), reaching 153 mg GAE g^−1^ DM. By contrast, raising *C*_CA_ to 15% did not provoke any significant increase in Y_TP_ compared to 10% (*p* > 0.05). This outcome evidenced that the optimum *C*_CA_ lay between 5 and 15%.

**Figure 3:**
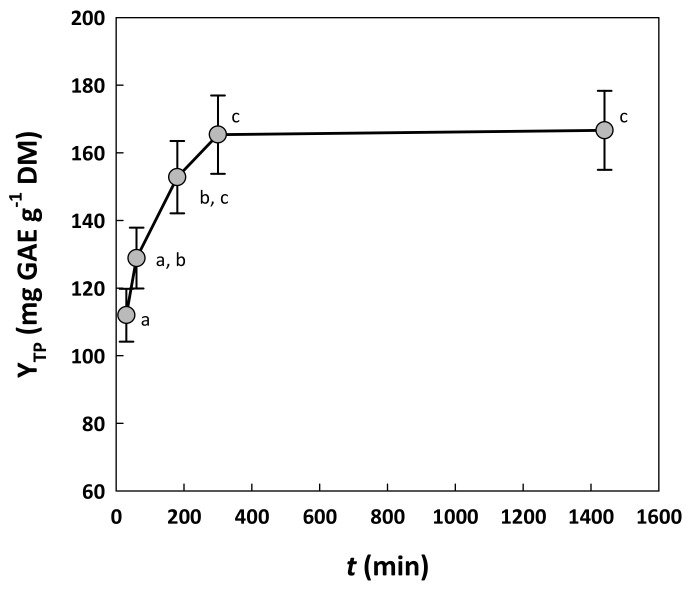
The effect of the residence time of the treatment on the yield in total polyphenols, at a constant temperature of 90 °C. Treatment was performed with a solvent system composed of 10% water, 60% glycerol and 30% ethanol, containing 10% citric acid. Points assigned with different letters (a, b, c) represent statistically different values (*p* < 0.05).

Finally, the residence time (t) was also assayed, to come up with a treatment period within which Y_TP_ could be maximized. To this end, extractions were performed with the mixture consisting of water/glycerol/ethanol (1/6/3), containing 10% citric acid. The time regime tested ranged from 30 to 300 min, based on preliminary experiments, but it was also extended up to 1440 min, to examine the effect of prolonged treatment (Figure 3). A relatively short residence time of up to 60 min was not favorable for achieving high Y_TP_, but statistically higher values reaching 165 mg GAE g^−1^ DM were obtained using a residence time of 300 min (*p* < 0.05). Higher Y_TP_ could not be attained, even when residence time was prolonged to 1440 min (24 h). Therefore, 300 min was selected as the most adequate upper value for testing the effect of residence time.

### 2.2. Severity-Based Modeling

Treatment severity is a measure of the harshness of the conditions used, which can be reflected in the severity factor. The pH of the treatment medium (solvent) is also considered an integral part of the severity [18]. Hence, the combined severity factor may be more descriptive in this regard, as it also takes into account the effect of solvent acidity (or alkalinity). Citric acid could act as a mild acid catalyst in lignocellulosic biomass disintegration [14,15], and the relevance of such an effect with polyphenol recovery merits investigation.

Considering that treatments were carried out at a constant temperature of 90 °C, the various severity levels tested were shaped by using different levels of acid catalysts and residence time. For each of those combinations, both CSF and CSF’ were determined and given along with the total polyphenol yield (Y_TP_) (Table 1). It was observed that the highest Y_TP_ (168 mg GAE g^−1^ DM) was achieved with a *C*_CA_ = 15% and t = 300 min. This value was significantly higher than those attained under any other set of conditions (*p* < 0.05), except the one achieved with a *C*_CA_ = 10% and t = 300 min (163 mg GAE g^−1^ DM). The difference in the severities of these two treatments was statistically non-significant, and thus the set of conditions *C*_CA_ = 10% and *t* = 300 min could be considered as the highest-performing treatment.

**Figure 4:**
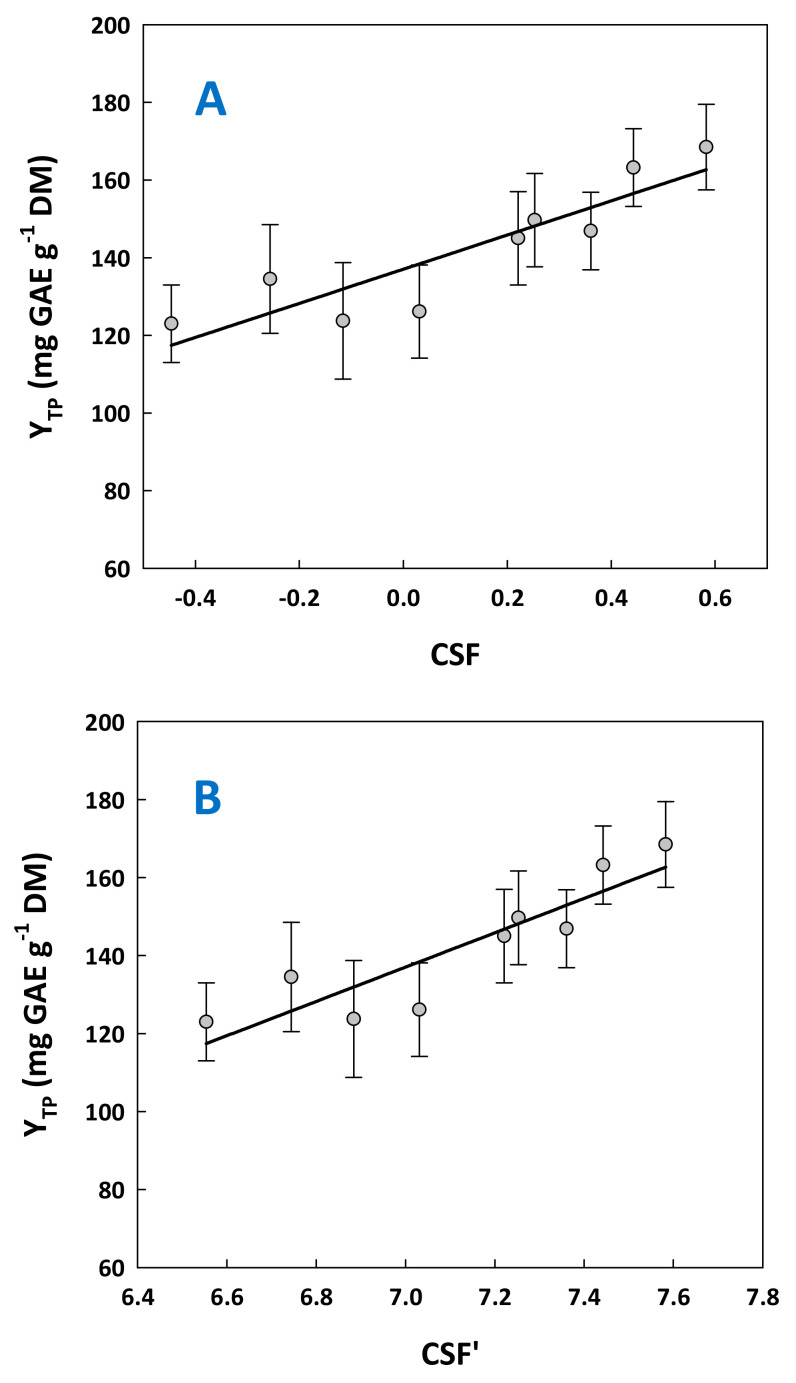
Correlation between the combined severity factor (**A**) and the alternative severity factor (**B**), with the yield in total polyphenols. Bars indicate standard deviation.

Another issue raised by the data obtained was the correlation between severity and total polyphenol yield. To examine such a hypothesis, Y_TP_ values were plotted as a function of both CSF and CSF’ (Figure 4). In both cases, Y_TP_ was found to be correlated with treatment severity in a linear manner, described by the following quadratic mathematical models:

Y_TP_ = 43.99CSF + 137.06 (R^2^ = 0.80, *p* = 0.0011) (1)

Y_TP_ = 43.99CSF’ − 170.87 (R^2^ = 0.80, *p* = 0.0011) (2)

Considering Equation (1), the theoretical value of Y_TP_ computed when CSF = 0.44 was 156 mg GAE g^−1^ DM. This value had no statistically significant difference with 163 mg GAE g^−1^ DM, determined experimentally. Likewise, using Equation (2) with a CSF’ = 7.44, the theoretical Y_TP_ equaled 156 mg GAE g^−1^ DM. Hence, both models could be used for fairly accurate predictions. On this ground, by adjusting residence time and citric acid concentration to provide a specific pH, the models represented by Equations (1) and (2) could provide approximate values for the yield in total polyphenols.

### 2.3. Response Surface-Based Modeling

**Figure 5:**
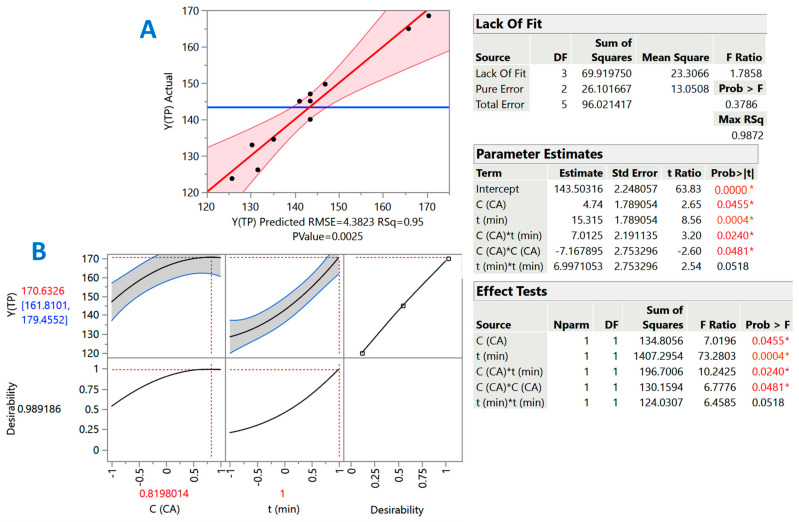
Diagram presenting the correlation between the predicted and actual values of the response (Y_TP_), obtained after implementing response surface methodology. The square correlation coefficient (R^2^) and the *p*-value for the model are also given (**A**). Diagram (**B**) shows the desirability factor, the maximum predicted Y_TP_, as well as the theoretical optimum *C*_CA_ and *t*. The inset tables contain the statistics associated with the response surface methodology. Values denoted with an asterisk are statistically significant (red: *p* < 0.05; orange, *p* < 0.001).

Taking into account the evidence that emerged from the severity-based models, the effects of the residence time (*t*) and acid catalyst (citric acid) concentration (*C*_CA_) were shown to be critical in maximizing total polyphenol recovery yield. Therefore, treatment optimization through the deployment of response surface methodology was considered as a suitable tool that could shed more light on the effect of these two variables. This approach aimed at evaluating the effects of *C*_CA_ and *t* on treatment performance but also to reveal any cross (synergistic) functions. Analysis of variance (ANOVA) and lack-of-fit tests were used to assess the validity of treatment modeling (Figure 5). The determined (measured) and predicted response (Y_TP_) values are shown in Table 2.

Any term of the model assessed as non-significant was excluded from the equation derived. Thus, the equation with only the significant terms (Figure 5, inset table “Parameter estimates”) was as follows:

Y_TP_ = 143.50 + 4.74X_1_ + 15.32X_2_ + 7.01X_1_X_2_ − 7.17X_1_^2^ (R^2^ = 0.95, *p* = 0.0025) (3)

**Figure 6:**
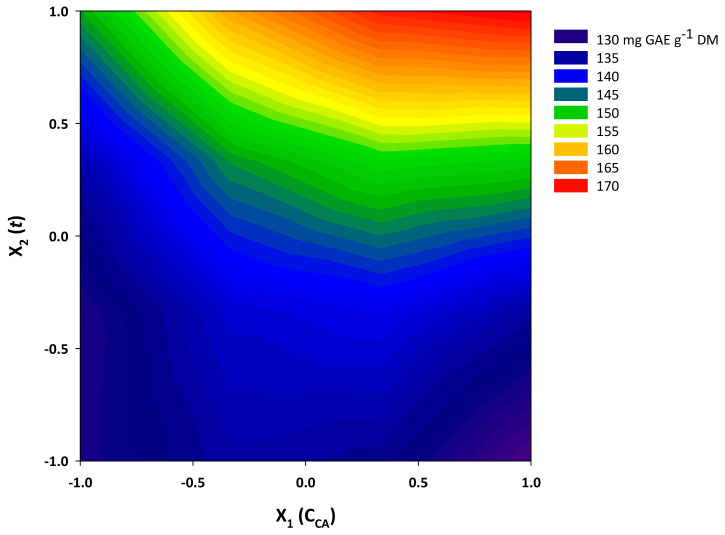
Contour plot displaying the yield in total polyphenols (Y_TP_) as a function of citric acid concentration (*C*_CA_) and residence time (*t*) variation.

Given the R^2^ and the significance of the *p*-value for lack of fit (confidence interval of at least 95%), it could be argued that the model had an excellent fitting to the experimental measurements. Furthermore, the Contour plot provided a visualization of the effect of the treatment variables on the response (Y_TP_) (Figure 6). The model (Equation (3)) constructed by implementing response surface methodology indicated that the effect of both treatment variables, *C*_CA_ (X_1_) and *t* (X_2_), was positive and statistically significant (*p* < 0.05). The synergistic effect between the two variables (X_1_X_2_) was also positive and significant (*p* < 0.05), but the quadratic effect of *C*_CA_ (X_1_) was negative (*p* < 0.05). This finding suggested that, beyond a certain limit, increases in citric acid concentration may not contribute to attaining higher Y_TP_. On the other hand, quadratic effects of *t* were non-significant (*p* > 0.05).

The predicted optimal values for both *C*_CA_ and *t* could be determined by the desirability function (Figure 5B), which also enabled the prediction of the maximum response. Thus, under optimal conditions estimated as *C*_CA_ = 14.1% and *t* = 300 min, the maximum Y_TP_ would be 171 ± 9 mg GAE g^−1^ DM. To investigate model applicability, three individual extractions were performed under the above-optimized conditions, giving a Y_TP_ value of 166 ± 6 mg GAE g^−1^ DM, which had no significant difference with the predicted maximum one (*p* < 0.05). This outcome affirmed the validity of the model.

### 2.4. Assessment of Treatment Performance

To shape a more integrated image of the polyphenolic load of the extract produced under optimized conditions, control extractions were also carried out using water, hydroethanolic and hydroglycerolic solvents, as proposed in a previous study [19], and the extracts were comparatively evaluated based on total polyphenol yield. However, because RGP extracts may be enriched in characteristic flavonoid classes, such as anthocyanin pigments and flavanols, additional indices were also considered, to have a wider approach regarding polyphenol recovery.

**Figure 7:**
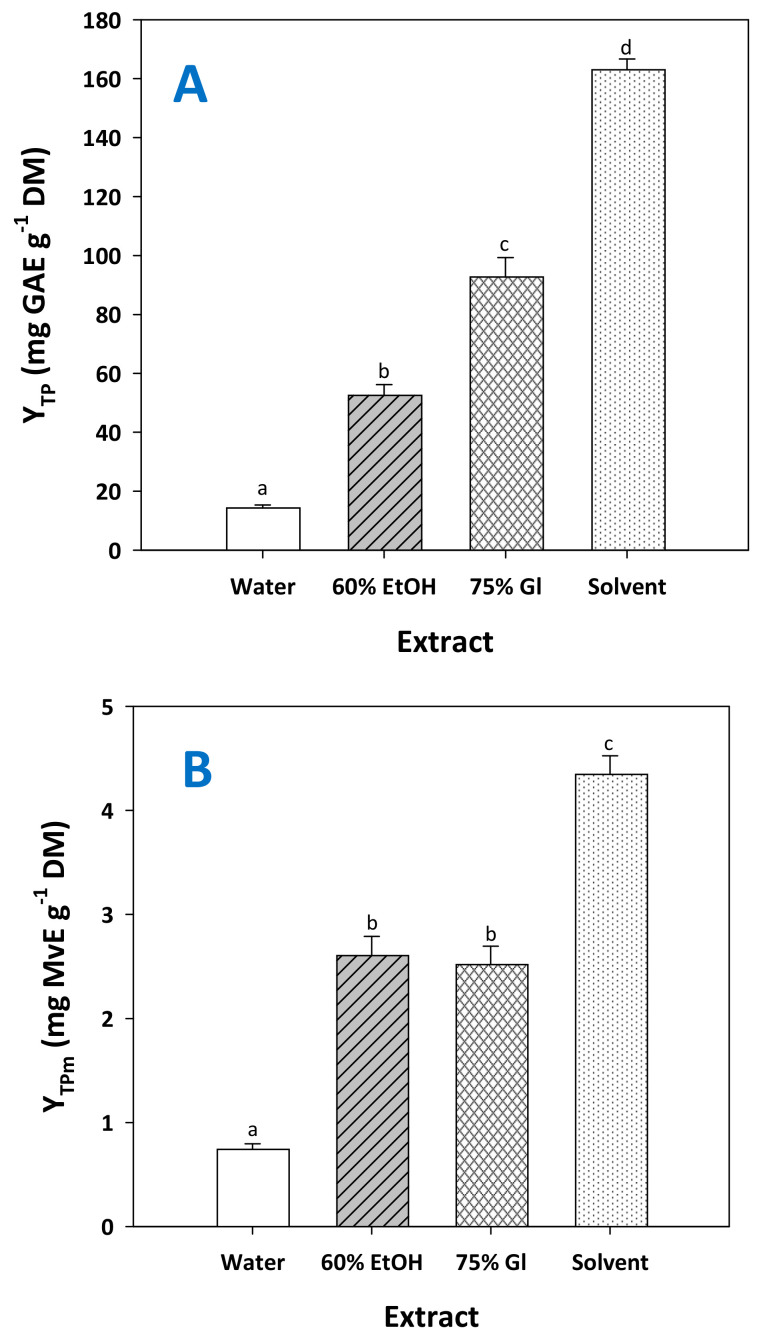

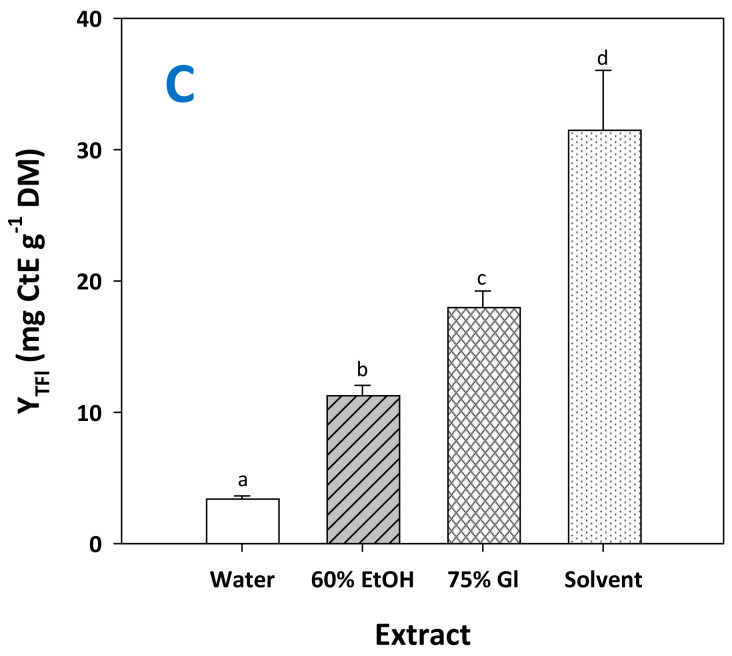
Yield in total polyphenols (**A**), total pigments (**B**) and total flavanols (**C**) achieved by treating RGP with the solvent, under optimized conditions (*C*_CA_ = 14.1%, t = 300 min). Columns assigned as 60% EtOH and 75% Gl correspond to treatments carried out with aqueous ethanol and aqueous glycerol. Assignments with different letters (a, b, c, d) signify statistically different values (*p* < 0.05).

The solvent with the optimized composition (water/ethanol/glycerol 1/3/6, 14.1% citric acid), assigned from now and on as “solvent”, was proven far more efficient than any other system tested, providing significantly higher total polyphenol yield (*p* < 0.05) (Figure 7A). Likewise, RGP treatment with the solvent produced extracts significantly enriched in total pigments (Figure 7B) and total flavanols (Figure 7C). These findings highlighted the effectiveness of the solvent in recovering polyphenols from major classes occurring in RGP. Furthermore, to have an insight into the capacity of the solvent, the results obtained were compared with selected results reported in the literature.

With respect to total polyphenols, conventional stirred-tank extraction with hydroethanolic solvent gave a yield in total polyphenols of 55 [20] and 68 mg GAE g^−1^ DM [21], while a combination of hydroethanolic solvent and microwave-assisted extraction afforded total polyphenol yield over 100 mg GAE g^−1^ DM [22]. Other authors used hydroethanolic solvent and ultrasound-assisted extraction and found yields that did not exceed 50 mg GAE g^−1^ DM, even after enzyme treatment [23]. The combination of hydroglycerolic solvent and ultrasonication gave a yield of 67 mg GAE g^−1^ DM [17]. On the other hand, yield levels as high as 142 mg GAE g^−1^ DM have been reported for exhaustive extraction of RGP with water/ethanol mixtures [24], but in similar processes, the yield was around 40 mg GAE g^−1^ DM [25]. An even higher yield of 154 mg GAE g^−1^ DM was attained with a water/acetone mixture [26].

A few studies have also brought out the role of acidification, which in some cases has been demonstrated to assist biomass organosolv treatment and recovery efficiency of polyphenols [27]. The alterations that the presence of acid could assist might be related to the efficient disintegration of the cellulose–hemicellulose–lignin complex, allowing easier and faster polyphenol entrainment into the solvent (liquid phase), as reported for glycerol-based organosolv treatment with oxalic [28], and formic-acid-catalyzed glycerol-based organosolv pretreatment [29]. Thus, a thorough investigation of various solvents for effective total polyphenol extraction from RGP showed that the addition of formic acid in water/methanol mixtures could significantly boost yield [30]. Similar results were reported for the RGP extraction with aqueous ethanol, acidified with HCl [31], but the use of citric acid as an acidifier has also been demonstrated to favor increased polyphenol recovery [32].

Although the type of the acid used, as well as acid concentration, have long before been known to exert a profound influence on pigment recovery from RGP [33], this was affirmed by later studies that demonstrated the effect of both citric and acetic acids [34]. On the other hand, acidification of water/methanol solvent with mineral acid (HCl) was shown to provide outstanding performance with regard to anthocyanin yield, which mounted up to 13.8 mg MvE g^−1^ DM [35]. Relatively high anthocyanin recovery has also been achieved with hydroethanolic solvent and conventional stirred-tank extraction [36], while other examinations performed with hydroethanolic solvent reported total anthocyanin yield as high as almost 21 mg MvE g^−1^ DM [37]. The above-mentioned data should be considered merely as indicative since extraction yield depends largely on the polyphenolic load of the RGP under examination. Thus, differences arising from genetic (varietal) factors of the grapes, as well as the vinification method, could have a prominent impact on the extraction yield.

### 2.5. Effect on Polyphenolic Composition and Antioxidant Characteristics

**Figure 8:**
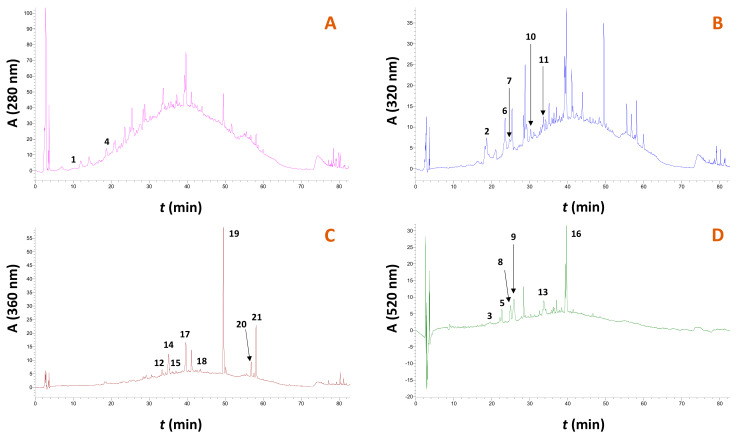
Representative chromatograms of the RGP extract obtained with treatment with the solvent, under optimized conditions. Chromatograms (**A**–**D**) were traced at 280, 320, 360 and 520 nm, respectively. Peak assignment: 1, gallic acid; 2, caftaric acid; 3, cyanidin 3-*O*-glucoside; 4, catechin; 5, delphinidin 3-*O*-glucoside; 6, p-coumarate derivative; 7, ferulate derivative; 8, petunidin 3-*O*-glucoside; 9, paeonidin 3-*O*-glucoside; 10, p-coumaric acid; 11, ferulic acid; 12, rutin; 13, malvidin 3-*O*-glucoside; 14, quercetin 3-*O*-glucuronide; 15, kaempferol 3-*O*-rutinoside; 16, malvidin 3-*O*-glucoside p-coumarate; 17, myricetin; 18, resveratrol; 19, quercetin; 20, kaempferol; 21, isorhamnetin.

Apart from the efficiency of the treatment in total polyphenol recovery, the analytical composition is of utmost importance. Therefore, HPLC analyses were carried out to profile the principal polyphenolic constituents of the extract obtained from the treatment with the solvent, and representative chromatograms are illustrated in Figure 8. Furthermore, to assess the ability of the solvent to extract polyphenols, analyses were also performed on the extracts generated with 60% ethanol, 75% glycerol and water, and the quantitative data are presented in Table 3.

In total, 21 compounds could be tentatively identified and quantified. Moreover, the extended raising in the baseline recorded at 280 and 320 nm witnessed the existence of a polymeric unresolved material, most probably composed of polymeric flavanols (condensed tannins). With reference to individual substances, malvidin 3-*O*-glucoside p-coumarate was predominant in all extracts analyzed, followed by paeonidin 3-*O*-glucoside and catechin. These results are in good agreement with those previously reported for Syrah pomace [38], Malbec pomace [39], and pomaces from various red varieties [30]. Selectivity of the solvent was seen for catechin, *p*-coumaric acid, ferulic acid, and delphinidin 3-*O*-glucoside, while the treatment with the solvent afforded extracts enriched in non-pigment polyphenols. On the other hand, treatment with 60% ethanol afforded extracts highly enriched in anthocyanin pigments, but also some flavonols including quercetin 3-*O*-glucuronide, myricetin and quercetin.

**Figure 9:**
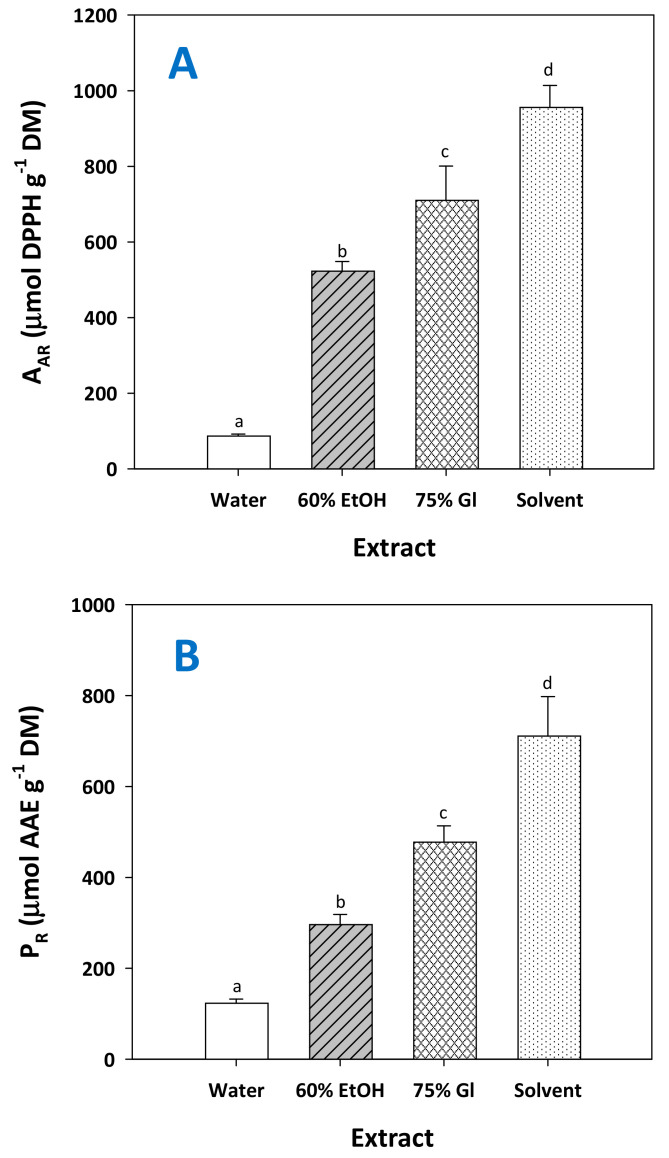
The antiradical activity (**A**) and the ferric-reducing power (**B**) of the extract generated after RGP treatment with the solvent, under optimized conditions (*C*_CA_ = 14.1%, *t* = 300 min). Columns assigned as 60% EtOH and 75% Gl correspond to treatments carried out with aqueous ethanol and aqueous glycerol. Assignments with different letters (a, b, c, d) signify statistically different values (*p* < 0.05).

The determination of the antioxidant activity of the extracts tested showed that the extract produced by the treatment with the solvent possessed significantly higher antiradical activity (A_AR_) and ferric-reducing power (P_R_) (Figure 9). This outcome was in line with the results of the total polyphenol yield (Figure 7A), suggesting that the most polyphenol-enriched extract expressed the strongest antioxidant effects. The richness in polyphenolic compounds has been correlated with enhanced antioxidant activity by several studies [37,40,41], yet a powerful antioxidant effect has also been ascribed to specific extract constituents. For example, exceptionally powerful antioxidant activity was found for grape seeds, and attributed to the high content of flavanols [42,43].

Studies on pure compounds have also shown that flavanols such as catechin may express higher antiradical activity compared to other polyphenols that occur in RGP [44]. On this ground, it could be argued that the higher A_AR_ and P_R_ displayed by the extracts obtained with solvent could be due to the increased concentration of catechin.

However, it should be emphasized that RGP extracts are particularly complex mixtures of polyphenols with variable antioxidant behavior, and therefore attribution of antioxidant effects to a single constituent might be an oversimplified and misleading approach. This is because previous examinations have provided strong evidence that, in polyphenol mixtures, the manifestation of antioxidant activity may be the integration of phenomena embracing synergism and antagonism [45,46]. Thus, the antioxidant activity estimated may well reflect such interactions.

## 3. Materials and Methods

### 3.1. Chemicals

Catechin hydrate (>98%), trans-resveratrol (>99%), quercetin 3-*O*-glucuronide (≥95%), cyanin chloride (≥90%), ferulic acid (99%), kaempferol, *p*-coumaric acid, kaempferol 3-*O*-rutinoside (98%), myricetin, isorhamnetin and rutin (quercetin 3-*O*-rutinoside) hydrate (>94%) were obtained from Sigma-Aldrich (Darmstadt, Germany). Sodium carbonate anhydrous (99%), Folin–Ciocalteu reagent, and glycerol anhydrous (99%) were purchased from Penta (Praha, Czechia). Formic acid (99%), L-ascorbic acid and citric acid (≥99.5%) were from Carlo Erba (Milan, Italy). All solvents used for chromatography were of appropriate (HPLC) grade. The radical probe 2,2-diphenyl-1-picrylhydrazyl (DPPH), quercetin hydrate (97%) and caffeic acid (99%) were from Alfa Aesar (Karlsruhe, Germany). Iron chloride hexahydrate (FeCl_3_ • 6H_2_O) was purchased from Merck (Darmstadt, Germany). 2,4,6-Tris(2-pyridyl)-s-triazine (TPTZ) was from Fluka (Steinheim, Germany).

### 3.2. Red Grape Pomace (RGP)

The pomace originated from the vinification of Syrah grapes (*Vitis vinifera* spp.), and it was collected after a typical red wine process that included a 7-day maceration. After collection, the pomace was transferred to the laboratory in air-tight plastic vessels within 48 h and freeze-dried for 24 h. The dried material was comminuted in a domestic table mill, and powder with a mean particle diameter < 300 μm was collected. This powder was stored in plastic containers at −40 °C and used for all experiments performed.

### 3.3. Extraction Procedures

Initially, screening extractions were performed with mixtures of water/ethanol/glycerol, where the percentage of water was kept constant at 10%, while ethanol and glycerol were used at various proportions. After establishing an optimum proportion of ethanol and glycerol, citric acid was added to the solvent at varying concentrations. In all these cases, 20 mL of solvent was transferred into a 25 mL Duran™ bottle with screw-cap closure and heated at a constant temperature of 90 °C, in an oil bath placed on a hotplate stirrer (Witeg, Wertheim, Germany). When the liquid acquired the temperature set, an exact mass of 1.00 g of dried, comminuted RGP was introduced into the bottle, and treatments were carried out under stirring at 500 rpm, for a residence time of 180 min. The treated samples were then cooled down and centrifuged at 10,000× *g*, to obtain a clear supernatant. In the cases of severity testing and response surface treatment optimization, the residence time and citric acid concentration varied according to the experimental design.

### 3.4. Determination of Treatment Severity

To test treatment severity on the performance of polyphenol recovery, different levels of severity were attained by properly adjusting residence time and citric acid concentration, which affected the pH of the solvent. Then, severity was determined as follows [47,48]:

(4) $$R_{o}=t\times e^{\left(\frac{T-100}{14.75}\right)}$$

SF = *logR*_o_ (5)

SF is termed as the severity factor, while *R*_o_ corresponds to severity. The value 100 is taken as the reference temperature (°C) and 14.75 is an empirical parameter associated with treatment temperature and activation energy.

The combined severity factor (CSF) represents an extended form of SF, taking into consideration the solvent pH, which may significantly affect biomatrix (RGP) decomposition [18]:


(6) $$R_{o}\prime =10^{-\mathrm{pH}}\times t\times e^{\left(\frac{T-100}{14.75}\right)}$$

CSF = *logR*_o_′ − pH (7)

An alternative expression of CSF, termed CFS’, has also been employed in previous examinations, and may be used to compare the severities of different treatments, within large pH ranges [18]:

CSF′
= *logR*_o_ + |pH − 7| (8)

### 3.5. Experimental Design for Treatment Optimization

Considering that all treatments took place under constant temperature (90 °C), two critical treatment factors (independent variables), the citric acid (catalyst) concentration (*C*_CA_) and time (*t*), were used to construct the design of experiment for the optimization, based on a response surface methodology. The deployment of a central composite design was chosen, encompassing 11 design points, 3 of them being central points of the experiment. The levels of both independent treatments (*C*_CA_, *t*) were codified in 3 levels, −1, 0 and 1, as described in an earlier study [49]. The codified and actual values of the variables may be seen in Table 4.

To select appropriate ranges for the values of both variables, single-factor experiments preceded the implementation of the experimental design. Modeling of the treatment was assessed by considering the overall significance (R^2^, *p*), while the significance of each of the models’ coefficients was used to omit non-significant factors from the equations (mathematical models). Assessment, as well as any other relevant statistical analysis, were accomplished with appropriate tests (lack-of-fit and ANOVA tests), taking 95% as the minimum significance.

### 3.6. Analyses for Total Polyphenols, Total Pigments, Total Flavanols and Antioxidant Properties

The analysis for the determination of total polyphenols was accomplished using a validated published methodology [50] and technical details reported elsewhere [51]. Gallic acid was used as the calibration standard, and results were calculated as gallic acid equivalents (GAE). Likewise, previously described protocols were used to determine both the antiradical activity (A_AR_) and the ferric-reducing power (P_R_) of the extracts produced [51]. A_AR_ and P_R_ were expressed as μmol DPPH per g dry mass (DM) and μmol ascorbic acid equivalents (AAE) per g DM, respectively. Total flavonols were analyzed following derivatization with *p*-dimethylaminocinnamaldehyde (DMACA) and expressed as catechin equivalents [34]. Total pigments were analyzed by implementing a previously reported methodology [52] and the results were reported as malvin (malvidin 3-*O*-glucoside) equivalents.

### 3.7. Chromatographic Analyses

The tentative identification of certain polyphenolic metabolites detected in RGP extracts was accomplished with liquid chromatography–diode array–mass spectrometry (LC–DAD–MS). More particularly, *trans*-caftaric acid was tentatively identified as described in an earlier study [53], using negative mode electrospray ionization. Similarly, an earlier methodology was deployed to identify major anthocyanins, using positive-mode electrospray ionization [54]. The chromatography device and the analytical settings used for quantitation were reported in detail in a previous study [51]. Quercetin 3-*O*-glucuronide, kaempferol 3-O-rutinoside, rutin, myricetin, kaempferol, gallic acid, *p*-coumaric acid, ferulic acid, quercetin, isorhamnetin and catechin were quantified with an external standard, using calibrations curves constructed with original standards of concentrations ranging from 0 to 50 μg mL^−1^. The *p*-coumarate and ferulate derivatives were quantified as *p*-coumaric acid and ferulic acid, respectively. Quantification of anthocyanins was accomplished using cyanin chloride as a standard and expressed as cyanin equivalents. Likewise, caffeic acid was used as a standard to quantify *trans*-caftaric acid. In all cases, the square correlation coefficient (R^2^) of the calibration curves was >0.999. All standard solutions were prepared in HPLC-grade methanol.

### 3.8. Data Handling and Statistics

The experimental design used for implementing response surface methodology, as well as the attendant statistics (ANOVA, lack of goodness of fit) and distribution analysis were performed with JMP™ Pro 16 (SAS, Cary, NC, USA). The linear and nonlinear regressions (significance level of at least 95%) were conducted with SigmaPlot™ 15.0 (Systat Software Inc., San Jose, CA, USA). The normality of the data was examined using the Shapiro–Wilk test. Since data were not found to be normally distributed, statistically significant differences were examined with the Kruskal–Wallis test, using IBM SPSS Statistics™ 29 (SPSS Inc., Chicago, IL, USA). Single-factor screening experiments and all organosolv treatments were carried out at least twice. Spectrophotometric and chromatographic analyses were performed in triplicate. The values presented are average ± standard deviation (SD).

## 4. Conclusions

The objective of this study was first the development of a novel tertiary solvent system for the effective recovery of red grape pomace polyphenols and then the implementation and optimization of an organosolv treatment of red grape pomace to maximize recovery. The assessment of the treatment using the severity factor showed that maximum performance may be achieved at nearly 1 maximum severity, within the limits tested. The following response surface-based optimization provided a model that predicted the ideal combination of citric acid concentration and time, to attain the highest polyphenol recovery. The extracts obtained using the optimized protocol exhibited significantly enhanced antioxidant properties, which might be of value in producing extracts destined for use as food antioxidants. Downstream processing to completely remove solvent could be bypassed, by removing only ethanol and water and obtaining virtually a glycerol-based extract. Such an approach could be a strategy for red grape pomace exploitation that should not be overlooked. Assuming that glycerol could be a part of the final product formulation, there could be a straightforward valorization of the extract. Such an option would be particularly appealing, considering that current trends dictate functionality-based and not purity-based ingredients. Thus, the extracts generated could be valorized for specific applications (e.g., food/cosmetic additives) rather than for general use. This concept might form the basis for the development of processes that could be scaled up, to produce neoteric industrial commodities through sustainable routes. Currently, work is in progress to appraise extract stability in glycerol-based matrices, which could further bring out the usefulness of the treatment proposed.

## Figures and Tables

**Table 1:** The effect of various combinations of citric acid concentration (*C*_CA_) and residence time (*t*) on the treatment severity (CSF and CSF’) and total polyphenol yield (Y_TP_).

*C*_CA_ (% *w*/*v*)	*t* (min)	CSF	CSF’	Y_TP_ (mg GAE g^−1^ DM)
5	60	−0.45 ^a^	6.55 ^a^	123.06 ^a^
	180	0.03 ^c^	7.03 ^c^	126.13 ^a^
	300	0.25 ^c^	7.25 ^c^	149.68 ^c^
10	60	−0.26 ^a^	6.74 ^a^	134.52 ^c^
	180	0.22 ^c^	7.22 ^c^	145.05 ^c^
	300	0.44 ^b^	7.44 ^b^	163.23 ^b^
15	60	−0.12 ^c^	6.88 ^c^	123.73 ^a^
	180	0.36 ^c^	7.36 ^c^	145.09 ^c^
	300	0.58 ^b^	7.58 ^b^	168.49 ^b^

Values within columns with different superscripted letters (a, b, c) are statistically different (*p* < 0.05).

**Table 2:** Response (Y_TP_) values corresponding to the points used to set up the experimental design.

Design Point	Independent Variables		Responses	
			Y_TP_ (mg GAE g^−1^ DM)	
	X_1_ (*C*_CA_, %)	X_2_ (*t*, min)	Measured	Predicted
1	−1 (5)	−1 (60)	133	130
2	−1 (5)	1 (300)	150	147
3	1 (15)	−1 (60)	124	126
4	1 (15)	1 (300)	168	170
5	−1 (5)	0 (180)	126	132
6	1 (15)	0 (180)	146	141
7	0 (10)	−1 (60)	135	135
8	0 (10)	1 (300)	165	166
9	0 (10)	0 (180)	145	144
10	0 (10)	0 (180)	147	144
11	0 (10)	0 (180)	140	144

**Table 3:** Analytical polyphenolic composition of the extract obtained after treatment with the solvent under optimized conditions and the extracts produced by the control treatments. Values represent means of triplicate determination (±standard deviation).

Compound	Yield (μg g^−1^ DM)			
	Water	60% EtOH	75% Gl	Solvent
Non-pigment polyphenols				
Gallic acid	9.1 ± 0.1 ^a^	nd	15 ± 2 ^b^	nd
Caftaric acid	41.9 ± 0.8 ^a^	29 ± 1 ^b^	44 ± 2 ^a, d^	40 ± 2 ^a, c^
Catechin	136 ± 8 ^a^	69 ± 3 ^b^	250 ± 10 ^c^	300 ± 20 ^d^
*p*-Coumarate derivative	14.6 ± 0.8 ^a^	nd	nd	16 ± 1 ^a^
Ferulate derivative	44 ± 1 ^a^	56 ± 2 ^b^	55 ± 3 ^b^	59 ± 6 ^b^
*p*-Coumaric acid	12.5 ± 0.8 ^a^	12.6 ± 0.5 ^a^	14.2 ± 0.4 ^b^	18 ± 2 ^c^
Ferulic acid	18 ± 1 ^a^	19 ± 2 ^a^	19.2 ± 0.9 ^a^	26.8 ± 0.4 ^b^
Rutin	6.4 ± 0.3 ^a^	16 ± 1 ^b^	11.4 ± 0.4 ^c^	16.5 ± 0.9 ^b^
Quercetin 3-*O*-glucuronide	47 ± 1 ^a^	99 ± 1 ^b^	78.9 ± 0.9 ^c^	83 ± 5 ^c^
Kaempferol 3-*O*-rutinoside	nd	6.7 ± 0.2 ^a^	2.29 ± 0.08 ^b^	3.5 ± 0.4 ^c^
Myricetin	17.5 ± 0.4 ^a^	92 ± 4 ^b^	45.0 ± 0.9 ^c^	50 ± 4 ^c^
Resveratrol	9.4 ± 0.1 ^a^	21.6 ± 0.8 ^b^	17.1 ± 0.2 ^c^	21 ± 1 ^b^
Quercetin	19.2 ± 0.2 ^a^	171 ± 5 ^b^	109 ± 4 ^c^	149 ± 9 ^d^
Kaempferol	7.8 ± 0.4 ^a^	32 ± 1 ^b^	18.9 ± 0.3 ^c^	30.8 ± 0.6 ^b^
Isorhamnetin	9.8 ± 0.1 ^a^	93 ± 1 ^b^	46.4 ± 0.4 ^c^	90 ± 5 ^b^
Total	394	718	730	901
Anthocyanin pigments				
Cyanidin 3-*O*-glucoside	24.2 ± 0.2 ^a^	27 ± 3 ^a^	5.3 ± 0.4 ^b^	13 ± 2 ^c^
Delphinidin 3-*O*-glucoside	65 ± 6 ^a^	78 ± 3 ^b^	61 ± 7 ^c^	115 ± 2 ^d^
Petunidin 3-*O*-glucoside	85 ± 5 ^a^	390 ± 24 ^b^	97 ± 8 ^a^	180 ± 10 ^c^
Paeonidin 3-*O*-glucoside	364 ± 4 ^a^	730 ± 40 ^b^	399 ± 6 ^c^	250 ± 20 ^d^
Malvidin 3-*O*-glucoside	36 ± 2 ^a^	131 ± 6 ^b^	65 ± 2 ^c^	74 ± 7 ^c^
Malvidin 3-*O*-glucoside *p*-coumarate	58 ± 1 ^a^	1320 ± 30 ^b^	315 ± 6 ^c^	420 ± 30 ^d^
Total	631	2676	942	1049
Sum	1025	3394	1671	1950

Values designated with different small letters (a, b, c, d) are statistically different (*p* < 0.05).

**Table 4:** The levels of citric acid concentration (*C*_CA_) and residence time (*t*) used for the experimental design, in their actual and codified forms.

Variable	Code	Levels		
		−1	0	1
*C*_CA_ (%)	X_1_	5	10	15
*t* (min)	X_2_	60	180	300

## Data Availability

The data presented in this study are available on request from the corresponding author. The data are not publicly available due to confidentiality required to continue the study.
